# Mesenchymal Stem Cells Inhibit Epithelial-to-Mesenchymal Transition by Modulating the IRE1*α* Branch of the Endoplasmic Reticulum Stress Response

**DOI:** 10.1155/2023/4483776

**Published:** 2023-07-26

**Authors:** Ruixi Luo, Yaqiong Wei, Peng Chen, Jing Zhang, La Wang, Wenjia Wang, Ping Wang, Weiyi Tian

**Affiliations:** ^1^Department of Immunology and Microbiology, School of Basic Medical Sciences, Guizhou University of Traditional Chinese Medicine, Guiyang, China; ^2^Stem Cell Therapy Research Center, Guizhou University of Traditional Chinese Medicine, Guiyang, China; ^3^Clinical Basis of Traditional Chinese Medicine Teaching and Research Section, School of Basic Medical Sciences, Guizhou University of Traditional Chinese Medicine, Guiyang, China; ^4^Institute of Experimental Animals, Guizhou University of Traditional Chinese Medicine, Guiyang, China

## Abstract

**Background:**

Idiopathic pulmonary fibrosis (IPF) is the most common idiopathic interstitial lung disease, and it carries a poor prognosis due to a lack of efficient diagnosis methods and treatments. Epithelial-mesenchymal transition (EMT) plays a key role in IPF pathogenesis. Endoplasmic reticulum (ER) stress contributes to fibrosis via EMT-mediated pathways. Mesenchymal stem cell (MSC) transplantation is a promising treatment strategy for pulmonary fibrosis and ameliorates lung fibrosis in animal models via paracrine effects. However, the specific mechanisms underlying the effect of transplanted MSCs are not known. We previously reported that MSCs attenuate endothelial injury by modulating ER stress and endothelial-to-mesenchymal transition. The present study investigated whether modulation of ER stress- and EMT-related pathways plays essential roles in MSC-mediated alleviation of IPF.

**Methods and Results:**

We constructed a A549 cell model of transforming growth factor-*β*1 (TGF-*β*1)-induced fibrosis. TGF-*β*1 was used to induce EMT in A549 cells, and MSC coculture decreased EMT, as indicated by increased E-cadherin levels and decreased vimentin levels. ER stress participated in TGF-*β*1-induced EMT in A549 cells, and MSCs inhibited the expression of XBP-1s, XBP-1u, and BiP, which was upregulated by TGF-*β*1. Inhibition of ER stress contributed to MSC-mediated amelioration of EMT in A549 cells, and modulation of the IRE1*α*-XBP1 branch of the ER stress pathway may have played an important role in this effect. MSC transplantation alleviated bleomycin (BLM)-induced pulmonary fibrosis in mice. MSC treatment decreased the expression of ER stress- and EMT-related genes and proteins, and the most obvious effect of MSC treatment was inhibition of the IRE1*α*/XBP1 pathway.

**Conclusions:**

The present study demonstrated that MSCs decrease EMT by modulating ER stress and that blockade of the IRE1*α*-XBP1 pathway may play a critical role in this effect. The current study provides novel insight for the application of MSCs for IPF treatment and elucidates the mechanism underlying the preventive effects of MSCs against pulmonary fibrosis.

## 1. Introduction

Idiopathic pulmonary fibrosis (IPF) is the most common idiopathic interstitial lung disease and is characterized by chronic, progressive worsening of pulmonary function. IPF has a poor prognosis, with a median survival of 2-3 years, due to a lack of efficient diagnosis methods and treatments [1]. IPF also imposes a heavy financial burden on patients and society because treatment costs ∼25,000 USD/person-year [2]. The triggers of IPF are not clear, and a multitude of factors, including smoking, environmental exposures, male gender, and aging, likely play a role [3].

A considerable number of studies have demonstrated that unrelieved alveolar injury and abnormal repair contribute to subsequent fibrosis [4]. During lung injury repair, continuous epithelial-mesenchymal transition (EMT) contributes to extracellular matrix accumulation and tissue remodeling [5]. EMT is the process by which epithelial cells lose tight junctions and apical-basal polarity and alter their shape from epithelial-like to mesenchymal-like under certain conditions [6]. EMT leads to fibrosis in a number of organs, including the kidney [7], liver [8], intestine [9], and lung. It has been widely reported and confirmed that EMT plays a role in the pathogenesis of IPF [10], and suppression of EMT alleviates IPF in animal models [11]. Many factors cause EMT, and activation of the unfolded protein response (UPR) and subsequent endoplasmic reticulum (ER) stress may contribute to fibrosis via EMT-mediated pathways [6]. Continuous ER stress is a causative factor of EMT in alveolar epithelial cells (AECs) exposed to ER stress inducers [12]. However, the precise underlying mechanisms among EMT, ER stress, and IPF are not clear.

Mesenchymal stem cells (MSCs) have become a focus of research in the cell therapy field due to their self-renewal and multilineage differentiation properties [13, 14]. Its powerful repair capacity gives it broad prospects for applications in the field of regenerative medicine [15]. In addition, MSCs have therapeutic potential for various diseases due to their immunomodulatory and paracrine properties [16]. Our previous studies found that MSCs have good efficacy in the treatment of diabetes mellitus [17], nonalcoholic fatty liver disease (NAFLD) [18], hyperuricemic nephropathy [19], and diabetic nephropathy [20] in animal models. Some studies have suggested that MSCs ameliorate bleomycin (BLM)-induced lung fibrosis via paracrine effects in mice [21, 22]. MSCs also reduce hypoxia-induced alveolar EMT *in vitro* by inhibiting the expression of transforming growth factor-*β*1 (TGF-*β*1) [23]. Stanniocalcin 1 secreted by MSCs reduces oxidative, ER stress, and the levels of profibrotic factors in AECs [24], but the specific molecular mechanism of these beneficial effects is not clear. We previously reported that MSCs attenuate endothelial-to-mesenchymal transition (EndMT) in endothelial cells by modulating ER stress [25]. Therefore, we asked whether there is a link between ER stress and EMT and hypothesized that modulation of ER stress and EMT-related pathways plays important roles in MSC-mediated alleviation of IPF. In the present study, we also investigated the regulatory effects of MSCs on ER stress and EMT in BLM-induced lung fibrosis in mice and the molecular mechanisms involved.

## 2. Materials and Methods

### 2.1. Cell Culture

A human AEC line (the A549 cell line) was kindly provided by Sichuan University and cultured in DMEM/F-12 (G4613, Servicebio, Wuhan, China) supplemented with 10% fetal bovine serum (FBS, Gibco, Waltham, MA, USA) at 37°C. Human umbilical cord-derived mesenchymal stem cells (huMSCs) were purchased from Zhongqiao Xinzhou Co., Ltd. (DF-GMP-ZB09BA, Shanghai, China). The certificate of analysis for the MSCs is provided in Annex S1. MSCs were cultured in MSC Complete Medium (ZQ-1320, Zhongqiao Xinzhou) supplemented with 5% serum substitute (ZQ-1320S, Zhongqiao Xinzhou) and antibiotics (S110JV, BasalMedia, Shanghai, China) at 37°C in 5% CO_2_. For the coculture experiments, huMSCs (passages 2–4) were seeded in a Transwell insert (Corning, New York, NY, USA) and cultured in huMSC Complete Medium overnight before being cocultured with A549 cells at a ratio of 2 : 5 in DMEM/F-12.

### 2.2. Cell Treatment

Untreated cells were used as controls. A549 cells were treated with TGF-*β*1 (10 ng/ml, 100-21, PeproTech, Rocky Hill, NJ, USA) for 72 hr with or without pretreatment with the ER stress inhibitor TUDCA (100 *μ*M, abs816166; Absin, Shanghai, China) or IRE1*α*-specific inhibitor 4 *μ*8c (10 *μ*M, T6363, Topscience, Shanghai, China) for 1 hr. Tunicamycin (TM, M4798, AbMole, Houston, TX, USA) was used to induce ER stress in A549 cells (0.5, 1, 2 and 5 *μ*M, 48 hr).

### 2.3. Cell Viability

Cell viability was measured using the Cell Counting Kit 8 (CCK8, CK04, Dojindo, Japan) according to the manufacturer's instructions. Briefly, the supernatant was removed, and CCK8 solution (diluted 1 : 10 in cell culture medium) was added to each well. The absorbance at 450 nm was measured using a microplate reader (Thermo Scientific, San Jose, CA, USA) after 2–4 hr.

### 2.4. Transmission Electron Microscopy (TEM)

A549 cells were prefixed and embedded with Electron microscope fixative (G1102, Servicebio, Wuhan, China). After the indicated treatments, the cells were observed under a transmission electron microscope (JEM-1400, Japan).

### 2.5. RNA Isolation and Quantitative Real-Time PCR (Q-PCR)

Total ribonucleic acid was extracted from A549 cells and lung tissues using TRIzol reagent (15596026, Thermo Scientific, USA) and reverse-transcribed into cDNA using a Reverse Transcription Kit (R312, Vazyme, Nanjing, China). Q-PCR was performed using SYBR Green qPCR Master Mix (B21203, Bimake, Houston, Texas, USA) on the CFX96 Real-Time PCR System (BioRad, Hercules, CA, USA). The primer information is listed in the Supplementary Table, and the primers for the reference gene (*β*-actin) were obtained from Sangon Biotech (Shanghai, China). The 2^−*ΔΔ*CT^ method was used to quantify relative gene expression.

### 2.6. Western Blotting

Total protein was extracted from A549 cells and lung tissues using radioimmunoprecipitation assay (RIPA) buffer (Beyotime, Shanghai, China). After the proteins were separated by sodium dodecyl sulfate-polyacrylamide gel electrophoresis, they were transferred to membranes, which were blocked with Protein Free Rapid Blocking Buffer (PS108, EpiZyme) and incubated with primary antibodies against CHOP (A5462, Bimake), BiP (11587-1-AP; Proteintech, Wuhan, China), ATF6 (D262665, Sangon, China), ATF4 (A5514, Bimake), XBP-1s (24868-1-AP, Proteintech), XBP-1u (25997-1-AP, Proteintech), IRE1*α* (A00683-1, Boster, Wuhan, China), phospho-IRE1*α* (S724, human) (ab124945, Abcam, Cambridge, UK), phospho-IRE1*α* (S724, mouse) (530878, ZEN-BIO, Chengdu, China), Vimentin (ET1610-39, HuaBio, Hangzhou, China), and E-cadherin (340341, ZEN-BIO) overnight. GAPDH (bs-0755R, Bioss, Beijing, China) was used as the internal reference protein. After incubation with HRP-conjugated goat anti-rabbit IgG (H + L) (AS014, ABclonal, Wuhan, China) and HRP-conjugated goat anti-mouse IgG (H + L) (AS003, ABclonal), the protein bands were visualized using a ChemiDoc™ Imaging System (Bio-Rad) and quantified using NIH ImageJ software (http://rsb.info.nih.gov).

### 2.7. Immunofluorescence

Immunofluorescence experiments were performed as previously described [22]. Lung tissue slices were incubated with anti-BiP (11587-1-AP, Proteintech) and anti-vimentin antibodies (BF8006, Affinity Biosciences, Changzhou, China). Slices containing climbing fibers were incubated with anti-vimentin (BF8006, Affinity Biosciences) and anti-E-cadherin (3195 T, CST, MA, USA) antibodies. After incubation with secondary antibodies and DAPI, the slices were observed under a fluorescence microscope (Olympus, Japan).

### 2.8. Animal Experiments

C57BL/6 mice were housed in the Animal Center of Guizhou University of Traditional Chinese Medicine in accordance with the Guide for the Care and Use of Laboratory Animals. Male wild-type C57BL/6 mice (8 weeks old, *n* = 15) were purchased from China Three Gorges University (SCXK (E) 2017-0012, Hubei, China). After a 1-week adaptation period, the mice were randomly assigned to the sham (control, *n* = 5), model (BLM, *n* = 5), and MSC treatment (BLM + MSC, *n* = 5) groups. The mice were anesthetized and intratracheally injected with 3.5 mg/kg BLM (B107423, Aladdin, Shanghai, China) to induce pulmonary fibrosis. The control mice received the same volume of saline. The animals were placed in a vertical position and subjected to rotation to ensure even distribution of the drug. One day after surgery, the mice in the BLM + MSC group were injected with mouse MSCs (passages 2–4, 5 × 10^5^ cells/mouse suspended in 200 *μ*L of PBS) via the tail vein every other week for a total of two times. Mouse MSCs were purchased from Procell Life Science & Technology Co., Ltd. (CP-M131, Wuhan, China). The mice in the control and BLM groups received the same volume of PBS. About 28 days after surgery, the mice were euthanized, and lung tissues and serum samples were harvested. The Institutional Animal Care and Use Committee of Guizhou University of Traditional Chinese Medicine approved all experimental procedures.

### 2.9. Hydroxyproline (HYP) Assay

The HYP content in the mouse lung was determined using a HYP assay kit (A030-2-1, NNJCBIO, Nanjing, China) according to the manufacturer's instructions. The HYP content is expressed in *μ*g/mg left lung (wet weight).

### 2.10. Histology

Mouse pulmonary tissues were fixed and embedded in paraffin. The tissues were sliced for hematoxylin and eosin (HE) staining (D006, NJJCBIO) and Masson's staining (D026, NJJCBIO). The slices were observed under a light microscope (Leica, Germany). The collagen area and alveolar space and number were quantified using ImageJ software.

### 2.11. Enzyme-Linked Immunosorbent Assay

The concentrations of IL-1*β* (70-EK201B, MultiSciences, Hangzhou, China), IL-6 (RK00008, ABclonal), TNF-*α* (SEKM-0034, Solarbio, Beijing, China), and TGF-*β*1 (SEKM-0035, Solarbio) in the serum were measured using ELISA kits according to the manufacturers' instructions.

### 2.12. Statistical Analysis

All experiments were performed at least three times. The data were analyzed using one-way analysis of variance (ANOVA) and Duncan's multiple-range test, Student's *t* test or the Bonferroni post hoc test. The data are presented as the means ± SEMs, and differences between experimental groups were considered statistically significant when *P* < 0.05.

## 3. Results

### 3.1. TGF-*β*1 Induced EMT in A549 Cells

**Figure 1:**
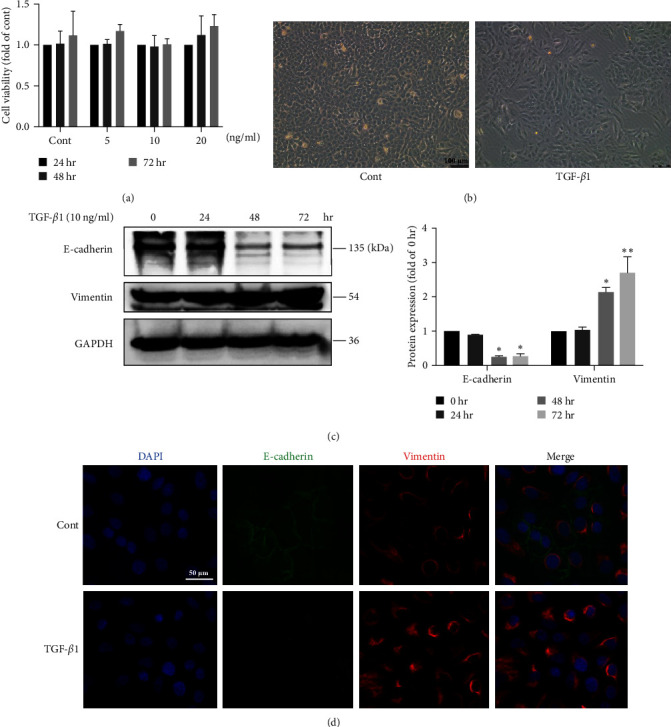
Transforming growth factor-*β*1 (TGF-*β*1) induced Epithelial–mesenchymal transition (EMT) in A549 cells. (a) Effects of TGF-*β*1 (5, 10, 20 ng/ml) on the viability of A549 cells after 24, 48 and 72 hr (*n* = 3, one-way ANOVA with Dunnett's test). (b) Phase-contrast images of A549 cells in the different groups. Scale bar, 100 *μ*m. (*n* = 3). (c) The protein expression levels of E-cadherin and vimentin were measured using western blotting, and the results were quantified using densitometry in ImageJ software (*n* = 3, one-way ANOVA with Duncan's post hoc test). (d) Images of immunofluorescence staining of E-cadherin (green) and vimentin (red) in A549 cells in the different groups. Scale bar, 50 *μ*m. The data are shown as the means ± SEM ( ^*∗*^*P* < 0.05,  ^*∗∗*^*P* < 0.01 vs. the control group).

TGF-*β*1 is commonly used to induce EMT in A549 cells. We first determined the cytotoxicity of TGF-*β*1 to A549 cells. A549 cells were exposed to different concentrations (5, 10, and 20 ng/ml) of TGF-*β*1 for 24, 48, or 72 hr. The CCK8 assay showed that TGF-*β*1 treatment did not significantly affect cell viability (Figure 1(a)). We treated A549 cells with 10 ng/ml TGF-*β*1 for 72 hr, and the cell morphology of A549 cells changed from an epithelial shape to a fibroblast-like shape (Figure 1(b)). The protein levels of the mesenchymal marker vimentin were markedly increased, and the level of the epithelial marker E-cadherin was decreased, which is consistent with the time course (Figure 1(c)). Immunofluorescence staining showed that vimentin expression was increased and E-cadherin expression was decreased in A549 cells after TGF-*β*1 treatment (Figure 1(d)). Furthermore, EMT-related markers including N-cadherin, TWIST1, Snail and Slug were also evaluated. The results showed that N-cadherin, TWIST1, Snail and Slug protein levels were all increased after TGF-*β*1 treatment (Figure S1). Collectively, these results indicated that TGF-*β*1 induced EMT in A549 cells.

### 3.2. MSCs Reduced EMT in A549 Cells

**Figure 2:**
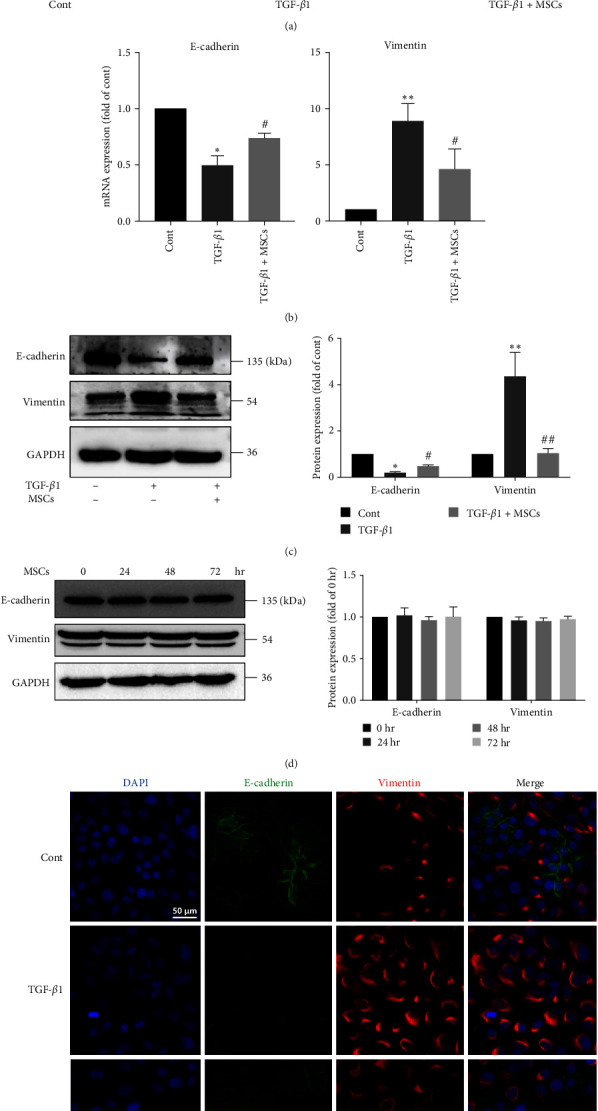
Mesenchymal stem cells (MSCs) ameliorated EMT in A549 cells. A549 cells were treated with 10 ng/ml TGF-*β*1 in the presence or absence of MSCs for 72 hr. (a) Representative phase-contrast microscopy images of A549 cells in the different groups. Scale bar, 100 *μ*m. (b) The mRNA expression levels of *E-cadherin* and *vimentin* were measured using Q-PCR (*n* = 3, one-way ANOVA with Duncan's post hoc test). (c) The protein expression levels of E-cadherin and vimentin were measured using western blotting, and the results were quantified using densitometry in ImageJ software (*n* = 4, one-way ANOVA with Duncan's post hoc test). (d) A549 cells were cocultured with MSCs in the absence of TGF-*β*1 for different times, and the protein expression levels of E-cadherin and vimentin were measured using western blotting. The results were quantified using densitometry in ImageJ software (*n* = 3, one-way ANOVA with Duncan's post hoc test). (e) Images of immunofluorescence staining of E-cadherin (green) and vimentin (red) in A549 cells in the different groups. Scale bar, 50 *μ*m. The data are shown as the means ± SEMs ( ^*∗∗*^*P* < 0.01,  ^*∗*^*P* < 0.05 vs. the control group; ^#^*P* < 0.05, ^##^*P* < 0.01 vs. the TGF-*β*1 group).

We examined whether MSCs can reduce EMT in A549 cells. Phase-contrast images revealed that A549 cells that were cocultured with MSCs maintained an epithelial shape after TGF-*β*1 treatment (Figure 2(a)). Q-PCR showed that the gene expression of E-cadherin was restored and the expression of vimentin was inhibited in the MSC group compared to the TGF-*β*1 group (Figure 2(b)). Western blot analysis also showed that MSCs restored the protein expression of E-cadherin and suppressed the expression of vimentin (Figure 2(c)). Coculture with MSCs did not influence the protein expression of EMT-related markers in A549 cells not treated with TGF-*β*1 (Figure 2(d)). Consistently, immunofluorescence staining showed that E-cadherin levels were significantly decreased and that vimentin protein expression was enhanced in A549 cells after TGF-*β*1 treatment and that coculture with MSCs reversed these alterations (Figure 2(e)). Furthermore, the results of western blot showed that MSCs significantly suppressed the expressions of N-cadherin, TWIST1, Snail and Slug (Figure S1). These findings suggested that MSCs reduced TGF-*β*1-induced EMT in A549 cells.

### 3.3. ER Stress Was Involved in TGF-*β*1-Induced EMT in A549 Cells

**Figure 3:**
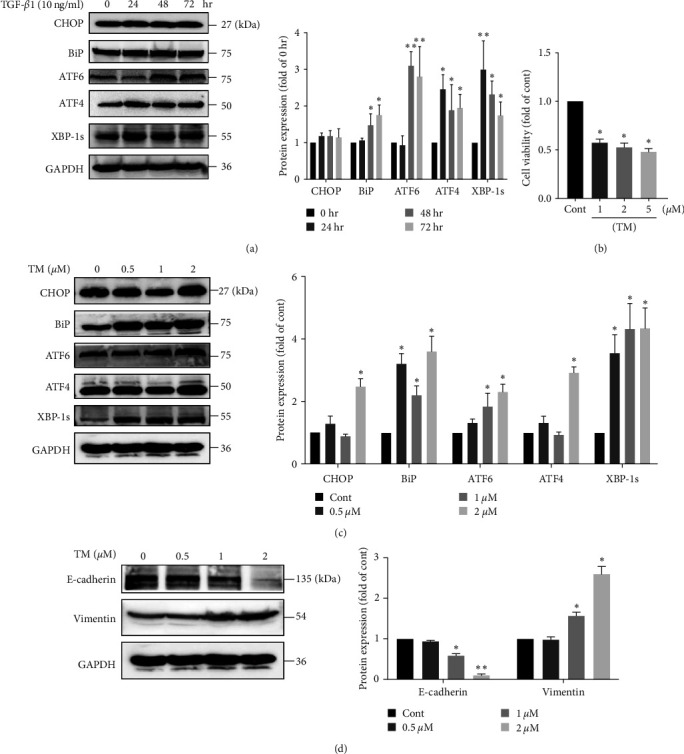
ER stress was involved in TGF-*β*1-induced EMT in A549 cells. (a) A549 cells were treated with 10 ng/ml TGF-*β*1 for different times. The protein expression levels of CHOP, BiP, ATF6, ATF4, and XBP-1s were measured using western blotting and quantified using densitometry in ImageJ software (*n* = 3, one-way ANOVA with Duncan's post hoc test). (b) Effects of TM (1, 2, and 5 *μ*M) on the viability of A549 cells after 48 hr (*n* = 3, one-way ANOVA with Dunnett's test). (c) A549 cells were treated with TM (0.5, 1 and 2 *μ*M) for 48 hr. The protein expression levels of CHOP, BiP, ATF6, ATF4, and XBP-1s were measured using western blotting and quantified using densitometry in ImageJ software (*n* = 3, one-way ANOVA with Duncan's post hoc test). (d) The protein expression levels of E-cadherin and vimentin were measured using western blotting and quantified using densitometry in ImageJ software (*n* = 3, one-way ANOVA with Duncan's post hoc test). The data are shown as the means ± SEMs ( ^*∗∗*^*P* < 0.01,  ^*∗*^*P* < 0.05 vs. the control group).

Increasing evidence indicates that ER stress drives EMT in different cellular systems [12]. Therefore, we tested whether TGF-*β*1 induces ER stress in A549 cells. We found that the protein levels of some ER stress markers, including ATF6, ATF4, XBP-1s, and BiP, were increased after treatment with 10 ng/ml TGF-*β*1 for different times (Figure 3(a)). Notably, TGF-*β*1 slightly upregulated CHOP expression (*P* > 0.05), which indicates that ER stress was not severe enough to induce apoptosis in A549 cells via CHOP-related pathway after TGF-*β*1 treatment for 72 hr. Next, we investigated whether ER stress induces EMT in A549 cells. A549 cells were cultured with different concentrations of a specific ER stress activator, TM (1, 2, or 5 *μ*M), for 48 hr, and it was found that cell viability was decreased by 50% in the TM group compared to the control group (Figure 3(b)). Then, A549 cells were treated with TM (0.50, 1, or 2 *μ*M) for 48 hr, and it was found that TM upregulated the protein expression of BiP, ATF4, ATF6, XBP-1s, and CHOP (Figure 3(c)). As expected, TM increased the expression of vimentin and decreased the expression of E-cadherin in A549 cells, which indicated the occurrence of EMT (Figure 3(d)). Collectively, these results demonstrated that ER stress was involved in TGF-*β*1-induced EMT in A549 cells.

### 3.4. MSCs Inhibited ER Stress in A549 Cells

**Figure 4:**
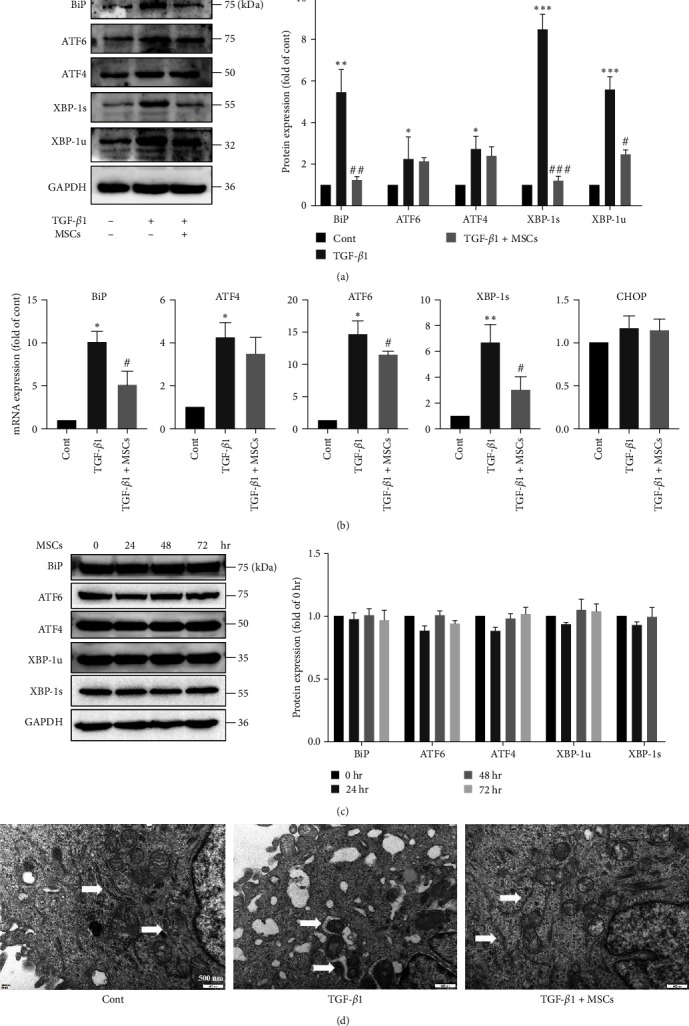
MSCs ameliorated ER stress in A549 cells. A549 cells were treated with 10 ng/ml TGF-*β*1 in the presence or absence of MSCs for 72 hr. (a) The protein expression levels of *BiP, ATF6, ATF4, XBP-1s and XBP-1u* were measured using western blotting and quantified using densitometry in ImageJ software (*n* = 3, one-way ANOVA with Duncan's post hoc test). (b) The mRNA expression levels of BiP, ATF4, ATF6, XBP-1s and CHOP were measured using Q-PCR (*n* = 3, one-way ANOVA with Duncan's post hoc test). (c) A549 cells were cocultured with MSCs in the absence of TGF-*β*1 for different times, and the protein expression levels of BiP, ATF6, ATF4, XBP-1s and XBP-1u were measured using western blotting. The results were quantified using densitometry in ImageJ software (*n* = 3, one-way ANOVA with Duncan's post hoc test). (d) Cellular ultrastructure in the different groups. The arrows indicate the ER. Scale bar, 500 nm. The data are shown as the means ± SEMs ( ^*∗∗∗*^*P* < 0.001,  ^*∗∗*^*P* < 0.01,  ^*∗*^*P* < 0.05 vs. the control group; ^###^*P* < 0.001, ^##^*P* < 0.01, ^#^*P* < 0.05 vs. the TGF-*β*1 group).

Our previous study showed that the MSCs ameliorate PA-induced ER stress in human umbilical vein endothelial cells [25]. Therefore, we investigated whether MSCs can inhibit ER stress in A549 cells. The results showed that MSCs significantly decreased the protein expression of XBP-1s, XBP-1u, and BiP, which was upregulated by TGF-*β*1, but MSCs did not obviously inhibit ATF4 or ATF6 expression in A549 cells (Figure 4(a)). Q-PCR also revealed that MSCs significantly decreased the expression of many ER stress-related genes, including *ATF6*, *XBP-1s*, and *BiP* (Figure 4(b)). Coculture with MSCs did not influence the protein levels of ER stress-related genes in A549 cells not treated with TGF-*β*1 (Figure 4(c)). To obtain more accurate results, TEM was performed. We found that the structure of the ER was ordered and tubular in A549 cells in the control group. However, the ER showed an irregular structure and was dilated after TGF-*β*1 treatment, and these changes were markedly improved by MSC coculture (Figure 4(d)). These results suggested that MSCs inhibited TGF-*β*1-induced ER stress in A549 cells.

### 3.5. Suppression of ER Stress Contributed to MSC-Mediated Amelioration of EMT in A549 Cells

**Figure 5:**
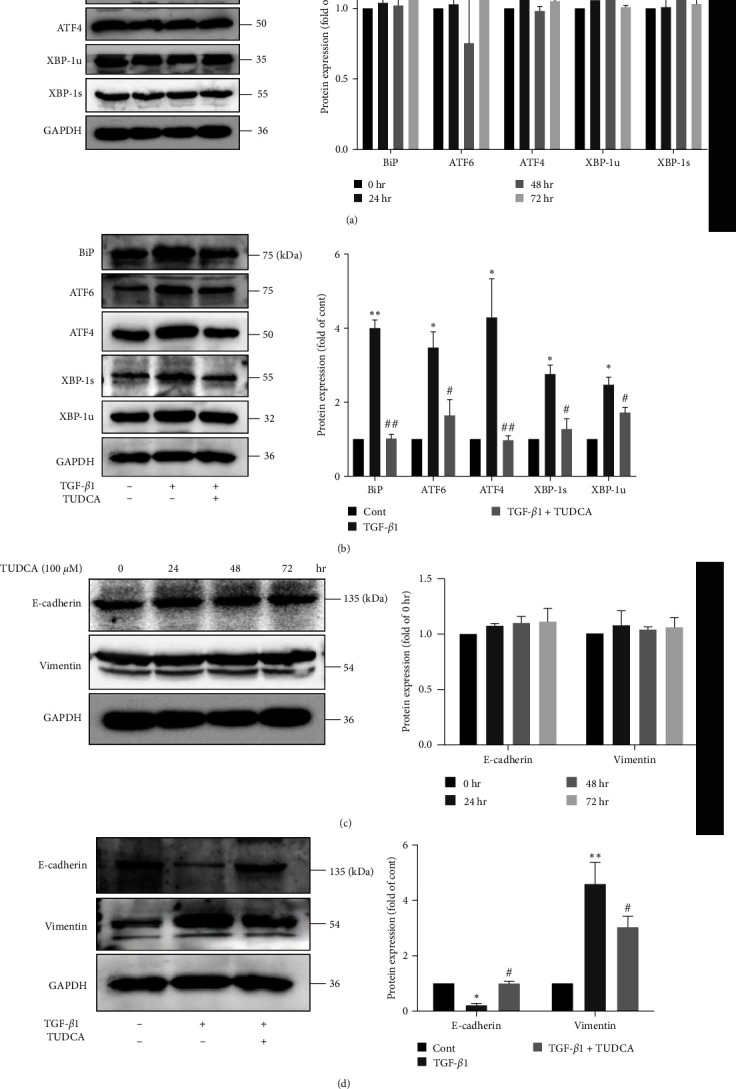
Suppression of ER stress contributed to MSC-mediated amelioration of EMT in A549 cells. (a) A549 cells were treated with 100 *μ*M TUDCA for different times. The protein expression levels of BiP, ATF6, ATF4, XBP-1s and XBP-1u were measured using western blotting and quantified using densitometry in ImageJ software (*n* = 3, one-way ANOVA with Duncan's post hoc test). (b) A549 cells were treated with 10 ng/ml TGF-*β*1 in the presence or absence of TUDCA for 72 hr. The protein expression levels of BiP, ATF6, ATF4, XBP-1s and XBP-1u were measured using western blotting and quantified using densitometry in ImageJ software (*n* = 4, one-way ANOVA with Duncan's post hoc test). (c) A549 cells were treated with 100 *μ*M TUDCA for different times. The protein expression levels of E-cadherin and vimentin were measured using western blotting and quantified using densitometry in ImageJ software (*n* = 3, one-way ANOVA with Duncan's post hoc test). (d) A549 cells were treated with 10 ng/ml TGF-*β*1 in the presence or absence of TUDCA for 72 hr. The protein expression levels of E-cadherin and vimentin were measured using western blotting and quantified using densitometry in ImageJ software (*n* = 4, one-way ANOVA with Duncan's post hoc test). The data are shown as the means ± SEMs ( ^*∗∗*^*P* < 0.01,  ^*∗*^*P* < 0.05 vs. the control group; ^##^*P* < 0.05, ^#^*P* < 0.05 vs. the TGF-*β*1 group).

To determine whether MSCs inhibit EMT by suppressing ER stress, we used a specific inhibitor of ER stress, TUDCA. TUDCA (100 *μ*M) alone did not influence the expression of ER stress-related proteins (BiP, ATF6, ATF4, XBP-1u, or XBP-1s) (Figure 5(a)) but significantly inhibited TGF-*β*1-induced ER stress in A549 cells (Figure 5(b)). Furthermore, the protein levels of E-cadherin were restored and the expression of vimentin was suppressed in the TUDCA group compared to the TGF-*β*1 group, which suggests that attenuation of ER stress helped reduce EMT (Figures 5(c) and 5(d)). These results demonstrated that attenuation of ER stress might contributed to MSC-mediated reduction of EMT in A549 cells.

### 3.6. MSCs Attenuated EMT via the IRE1*α*/XBP1 Pathway

**Figure 6:**
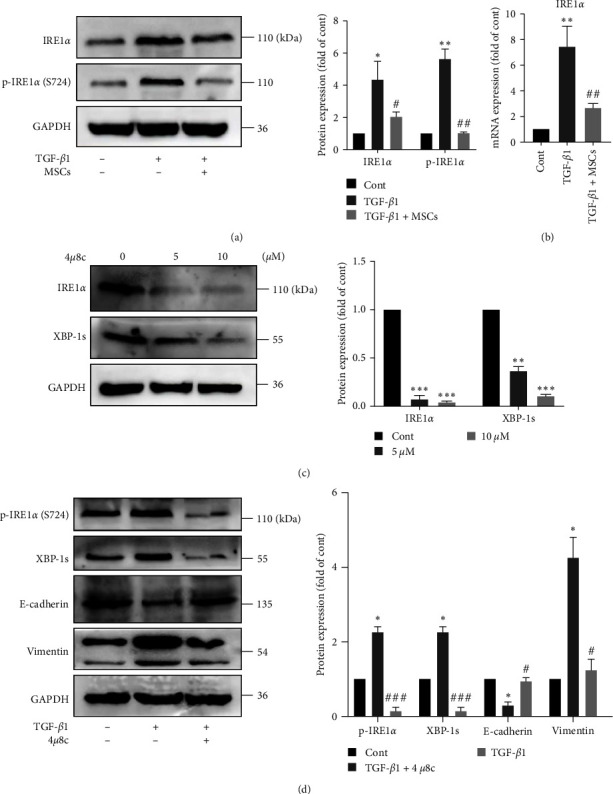
MSCs attenuated EMT via the IRE1*α*/XBP1 pathway. (a) The protein expression levels of IRE1*α* and p-IRE1*α* were measured using western blotting and quantified using densitometry in ImageJ software (*n* = 4, one-way ANOVA with Duncan's post hoc test). (b) The mRNA expression level of IRE1*α* was measured using Q-PCR (*n* = 3, one-way ANOVA with Duncan's post hoc test). (c) A549 cells were treated with 4 *μ*8c (5 and 10 *μ*M) for 48 hr, and the protein expression levels of IRE1*α* and XBP-1s were measured using western blotting and quantified using densitometry in ImageJ software (*n* = 4, one-way ANOVA with Duncan's post hoc test). (d) A549 cells were treated with 10 ng/ml TGF-*β*1 in the presence or absence of 4 *μ*8c for 72 hr. The protein expression levels of p-IRE1*α*, XBP-1s, E-cadherin and vimentin were measured using western blotting and quantified using densitometry in ImageJ software (*n* = 4, one-way ANOVA with Duncan's post hoc test). The data are shown as the means ± SEMs ( ^*∗∗∗*^*P* < 0.001,  ^*∗∗*^*P* < 0.01,  ^*∗*^*P* < 0.05 vs. the control group; ^###^*P* < 0.001, ^##^*P* < 0.01, ^#^*P* < 0.05 vs. the TGF-*β*1 group).

The ER stress response comprise three signaling branches: the ATF6, PERK, and IRE1*α* branches.  Figure 1 shows that MSCs particularly inhibited XBP-1s protein expression in A549 cells. Therefore, we hypothesized that the IRE1*α*/XBP1 pathway plays an important role in MSC-mediated reduction of EMT in A549 cells. To test this hypothesis, we first examined the expression of IRE1*α*, an upstream signaling molecule of XBP1. Western blot analysis showed that TGF-*β*1 prominently upregulated the expression of IRE1*α* and phosphorylated (p)-IRE1*α* (S724) and that MSC coculture suppressed IRE1*α* and p-IRE1*α* protein expression (Figure 6(a)). Similar results were obtained using Q-PCR (Figure 6(b)). To verify whether MSCs inhibit EMT by inhibiting the IRE1*α*-XBP1 pathway in A549 cells, we used 4 *μ*8c, a specific inhibitor of IRE1*α* activity (Figure 6(c)). The results showed that 4 *μ*8c (10 *μ*M) treatment attenuated the TGF-*β*1-induced alterations in EMT-related protein expression (Figure 6(d)). Overall, our study indicated that the IRE1*α*-XBP1 pathway is at least partially associated with MSC-mediated reduction of EMT in A549 cells.

### 3.7. MSCs Ameliorated BLM-Induced Pulmonary Fibrosis in Mice

**Figure 7:**
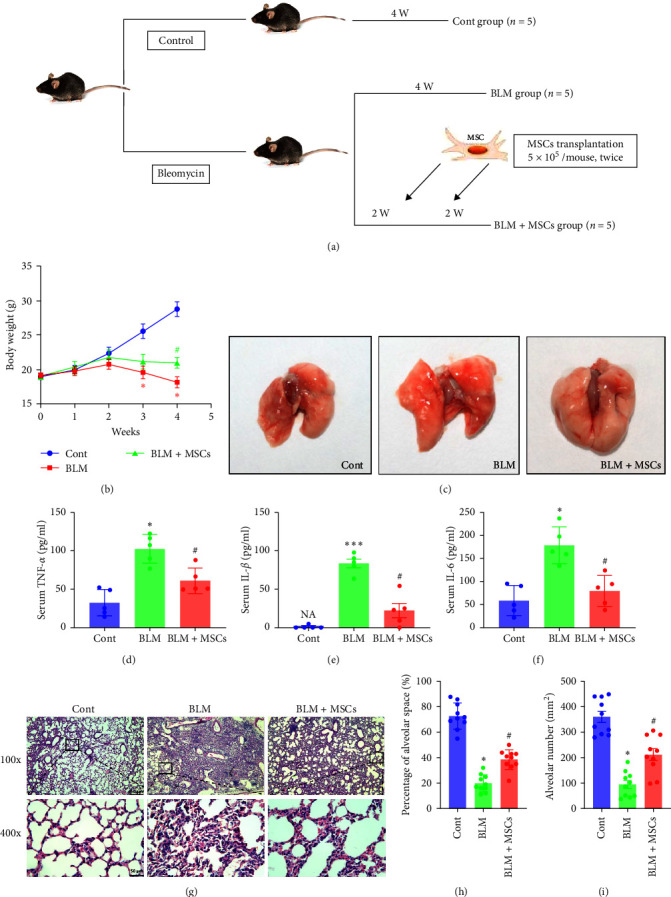
MSCs alleviated BLM-induced pulmonary injury in mice. (a) Diagram of the groups for the *in vivo* experiments. (b) Body weight curves of mice in the different groups (*n* = 5, Student's *t* test). (c) The overall appearances of the left and right lungs of mice from the anterior view. (d–f) Serum levels of TNF-*α*, IL-1*β* and IL-6 were measured after 4 weeks (*n* = 5, Student's *t* test). (g) Representative HE-stained images of mouse lung tissues. Scale bar, 250 *μ*m (100x), 50 *μ*m (400x). (h–i) The percentage of alveolar space and alveolar number were analyzed in ImageJ (*n* = 10, one-way ANOVA with the Bonferroni post hoc test). The data are shown as the means ± SEMs ( ^*∗∗∗*^*P* < 0.001,  ^*∗*^*P* < 0.05 vs. the control group; ^#^*P* < 0.05 vs. the BLM group).

The results presented above show that MSCs attenuate EMT by regulating ER stress *in vitro*. We performed follow-up *in vivo* experiments to confirm the effects of MSCs (Figure 7(a)). Intratracheally, injection of bleomycin is commonly used as an experimental model of lung fibrosis. In this study, we built lung fibrosis model in mice as previously reported [23]. During the study, no mice in any of the three groups died. About 28 days after the initial surgery, BLM treatment decreased body weight, and MSC transplantation reversed this effect (Figure 7(b)). However, no morphological differences were observed in the lung tissues of mice in any of the groups (Figure 7(c)). The serum levels of TNF-*α*, IL-1*β*, and IL-6 were lower in the BLM + MSC group compared with the BLM group, which indicated that MSCs reduced inflammation in mice with lung fibrosis (Figure 7(d)–7(f)). HE staining revealed that the pulmonary alveolar space and number were obviously decreased, the amount of connective tissue was markedly increased, and the alveolar wall was thickened in mice that received BLM. The alveolar space and number were significantly increased and the amount of connective tissue was significantly decreased in the BLM + MSC group compared to the BLM group (Figure 7(g)–7(i)).

**Figure 8:**
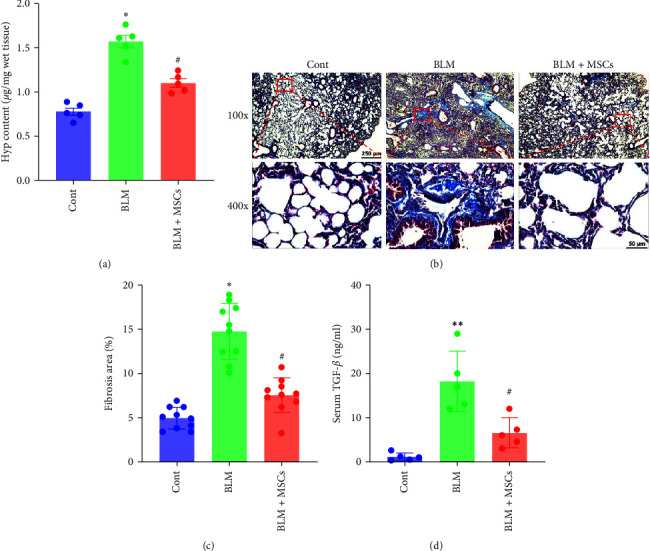
MSCs ameliorated BLM-induced pulmonary fibrosis in mice. (a) HYP content in lung tissue was assessed (*n* = 5, one-way ANOVA with the Bonferroni post hoc test). (b) Representative Masson's trichrome-stained images of the lung tissues of mice. Scale bar, 250 *μ*m (100x), 50 *μ*m (400x). (c) The fibrosis area was analyzed in ImageJ (*n* = 10, one-way ANOVA with the Bonferroni post hoc test). (d) The serum levels of TGF-*β*1 were measured after 4 weeks (*n* = 5, Student's *t* test). The data are shown as the means ± SEMs ( ^*∗∗*^*P* < 0.01,  ^*∗*^*P* < 0.05 vs. the control group; ^#^*P* < 0.05 vs. the BLM group).

Next, pulmonary fibrosis was assessed. HYP levels were measured to assess the collagen content in the lung. The HYP content was significantly increased in the BLM group compared to the control group, and MSCs decreased the HYP level (Figure 8(b)). Masson trichrome staining showed that the collagen levels were increased in the alveolar and interstitial regions in the BLM group compared to the control group, whereas collagen deposition was markedly decreased in the MSC treatment group (Figures 8(b) and 8(c)). The serum level of TGF-*β*1 was significantly increased in BLM-treated mice upon the development of lung fibrosis and was decreased by MSC transplantation (Figure 8(d)). These results indicated that MSCs alleviated BLM-induced pulmonary fibrosis in mice.

### 3.8. MSCs Inhibited ER Stress and EMT in the Lungs of Mice with Lung Fibrosis

**Figure 9:**
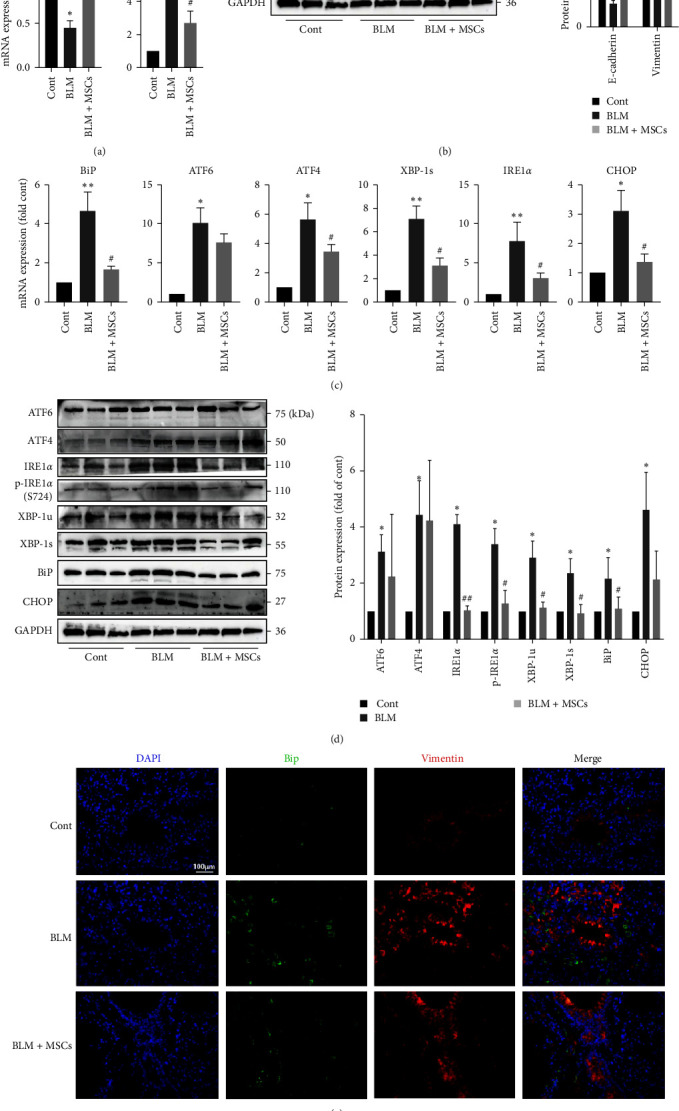
MSCs attenuated ER stress and EMT in the lungs of mice with lung fibrosis. (a) The mRNA expression levels of *E-cadherin* and *Vimentin* in lung tissues were measured using Q-PCR (*n* = 5, one-way ANOVA with Duncan's post hoc test). (b) The protein expression levels of E-cadherin and vimentin in lung tissues were measured using western blotting, and the results were quantified via densitometry by using ImageJ software (*n* = 3, one-way ANOVA with Duncan's post hoc test). (c) The mRNA expression levels of *Bip*, *Atf6*, *Atf4*, *Xbp-1s*, *Ire1α* and *Chop* in lung tissues were measured using Q-PCR (*n* = 5, one-way ANOVA with Duncan's post hoc test). (d) The protein expression levels of ATF6, ATF4, IRE1*α*, p-IRE1*α*, XBP-1s, XBP-1u, BiP and CHOP in lung tissues were measured using western blotting, and the results were quantified via densitometry by using ImageJ software (*n* = 3, one-way ANOVA with Duncan's post hoc test). (e) Images of immunofluorescence staining of BiP (green) and vimentin (red) in the lung tissues of mice. Scale bar, 100 *μ*m. The data are shown as the means ± SEMs ( ^*∗∗*^*P* < 0.01,  ^*∗*^*P* < 0.05 vs. the control group; ^#^*P* < 0.05, ^##^*P* < 0.01 vs. the BLM group).

We first assessed whether MSC transplantation decreases EMT in lung tissues. We analyzed EMT by measuring E-cadherin and vimentin levels. Q-PCR and Western blotting showed that E-cadherin expression was decreased and vimentin expression was increased in BLM-treated mice and that these effects were reversed by MSC treatment (Figures 9(a) and 9(b)). The levels of ER stress markers were evaluated. MSC treatment markedly decreased the expression of several ER stress-related genes (*Atf4*, *Ire1α*, *Xbp-1s*, *Bip*, and *Chop*) in lung tissues (Figure 9(c)). However, the protein expression of ATF6, IRE1*α*, p-IRE1*α* (S724), XBP-1s, XBP-1u, BiP, and CHOP was upregulated in the lung tissues of BLM-treated mice and decreased after MSC transplantation (Figure 9(d)). Notably, the most obvious effect of MSC treatment was inhibition of the IRE1*α*/XBP1 pathway. To more intuitively observe ER stress and EMT in lung tissues, dual immunofluorescence staining of BiP and vimentin was performed. As shown in Figure 9(e), a significant increase in BiP and vimentin expressed was observed in lung tissues in the BLM group, and this increase was attenuated in the BLM + MSC group. Taken together, these results indicated that MSCs attenuated ER stress and EMT in the lungs of mice with lung fibrosis and that suppression of the IRE1*α*/XBP1 pathway may play a critical role in this effect.

## 4. Discussion

The present research examined the effects and underlying molecular mechanisms of MSCs in *in vitro* and *in vivo* models of IPF. We found that MSCs decreased TGF*β*1-induced EMT by inhibiting ER stress in A549 cells. We showed that the IRE1*α*/XBP-1 pathway may play an important role in MSC-mediated reduction of EMT. We also confirmed that these results in a BLM-induced IPF mouse model (Figure S2).

The pathogenesis of IPF is not fully understood, and several factors likely to play a role. Chronic AEC injury deserves attention. There are two types of AECs: type I (AT-I) cells and type II (AT-II) cells. AT-I cells execute gas exchange, and AT-II cells serve as progenitor cells and differentiate into AT-I cells after injury [26]. Currently, it is believed that AECs play a central role in the pathogenesis of IPF due to their abnormal regenerative function. Chronic AEC injury may result from various continuous stimuli, such as inflammation, stress, pollutants, and infection [27]. Aberrant alveolar repair and an imbalance between profibrotic and antifibrotic mediators lead to IPF [28]. Increasing evidence shows that EMT contributes to promoting the formation of profibrotic microenvironment via dysregulation of paracrine signaling in AT-II cells [29]. Therefore, we focused our studies on EMT. TGF*β* is one of the most powerful cytokines that drive fibrosis, and it is widely used to induce EMT in AECs [30]. In the present study, A549 cells were treated with TGF-*β*1 for 72 hr to induce EMT *in vitro*. The results showed that the protein level of vimentin was increased and E-cadherin was decreased after TGF-*β*1 treatment. In addition, the cell morphology of A549 cells changed from an epithelial shape to a fibroblast-like shape. Therefore, we confirmed that the EMT *in vitro* model was established in our study. Subsequent experiments showed that MSCs reduced EMT in A549 cells. Notably, many studies have reported that MSCs promote EMT and increase the migration and invasion of A549 cells [31]. Other studies have shown that MSCs prevent EMT in TGF-*β*-treated A549 cells. The inconsistencies between these studies are likely attributable to the different sources of MSCs, the different coculture systems (e.g., the cell number and proportion) and the variability in the method of cell isolation. Beane OS reported that bone-marrow-derived MSCs (BM-MSCs) from aging donors show abnormal function compared to MSCs derived from younger donors [32]. Therefore, establishing a clear and intact evaluation system for MSCs is critical.

Under normal conditions, protein homeostasis is maintained by the balance between protein synthesis and degradation [33]. However, the UPR is activated when unfolded and misfolded proteins accumulate in the ER. The UPR is a cytoprotective response aimed at restoring protein homeostasis, but an excessive or prolonged UPR results in ER stress [34]. Increased ER stress is a causative factor of EMT in AECs exposed to ER stress inducers [12]. Our previous study showed that ER stress inducers (TM and thapsigargin) induce EndMT in HUVECs [25]. Therefore, we evaluated ER stress in TGF-*β*1-treated A549 cells. ER stress involves three signaling pathways: the PERK/ATF4, ATF6, and IRE1*α*/XBP1 pathways. Therefore, we measured the protein expression of ATF4, ATF6, XBP-1s, BiP, and CHOP. TGF-*β*1 upregulated the protein expression of ATF4, ATF6, XBP-1s, and BiP, which indicated that ER stress occurred in A549 cells. Notably, the expression of CHOP, which mediates ER stress-induced apoptosis, was not increased (*P* > 0.05). In contrast, we noticed that palmitic acid robustly increased the expression of CHOP and other ER stress-related proteins in HUVECs, and cell viability was significantly decreased after 24 hr in our previous study [25]. TGF-*β*1 did not decrease cell viability after 72 hr. Therefore, we speculate that TGF-*β*1-induced EMT is a less severe process that does not induce ER stress-mediated apoptosis.

We also treated A549 cells with the ER stress inducer TM. TM upregulated the expression of ATF4, ATF6, XBP-1s, and CHOP, and also induced EMT in A549 cells. Therefore, we conclude that ER stress is involved in TGF-*β*1-induced EMT. However, multiple signaling pathways, including classic pathways such as the Smad, PI3K/AKT, and RhoA/ROCK pathways, are involved in TGF-*β*1-induced EMT in A549 cells [35]. We focused our attention on ER stress because of the high anti-ER stress capacity of MSCs, which we previously reported, and we presumed that ER stress was a key target of MSC-mediated amelioration of EMT. Surprisingly, MSCs markedly suppressed the protein expression of XBP-1s and XBP-1u, which prompted us to analyze the expression of molecules upstream of XBP1 signaling, that is, IRE1*α*/p-IRE1*α* (S724). MSC coculture obviously suppressed IRE1*α* and p-IRE1*α* mRNA and protein expression. Inhibition of IRE1*α* activity using the IRE1 RNase inhibitor 4 *μ*8c produced similar results, which indicates that the modulation of the IRE1/XBP1 pathway may be an important mechanism by which MSCs protect against TGF-*β*1-induced EMT. However, MSCs exert their beneficial effects in numerous ways. Therefore, it is difficult to conclude that modulation of the IRE1/XBP1 pathway is the most important mechanism underlying the protective effects of MSCs. We observed that 4 *μ*8c had a less robust protective effect against EMT in A549 cells than MSCs. Therefore, we speculate that the pathways involved in the protective effect of MSCs are multifaceted, and further studies are needed.

To further verify the *in vitro* results, we constructed a BLM-induced IPF mouse model. MSCs were reported to be distributed mainly in the lungs after intravenous injection [36]. In our previous study, we transplanted PKH26-labeled MSCs into rats and found that MSCs mainly stayed in the lung, liver and kidney for 7 days [18]. In the present study, we also infused RFP-labeled MSCs through tail vein to mice and found the fluorescent signal existed in lung until 7 days (data not shown). Therefore, we speculated that MSCs was able to exert its beneficial paracrine effect in lungs, Several recent studies have reported the protective effects of MSCs in animal models of IPF [37–39]. However, few studies have focused on the effects of MSCs on ER stress and EMT in BLM-treated mice. To the best of our knowledge, the current study is the first to comprehensively assess the contribution of the three signaling branches to the UPR. The results showed that MSCs specifically inhibited the IRE1*α*/XBP1 pathway in an IPF mouse model. These results are consistent with the *in vitro results*. Therefore, suppression of the IRE1*α*/XBP1 pathway is likely key for MSC-mediated amelioration of EMT.

In conclusion, we demonstrated that MSCs ameliorated TGF*β*1-induced EMT by modulating ER stress in A549 cells and that blockade of the IRE1*α*-XBP1 pathway was associated with these effects. MSCs attenuated ER stress and EMT in the lungs *in vivo*, which contributed to the amelioration of lung fibrosis in BLM-treated mice. The current study provides novel insight for the application of MSCs for the treatment of IPF and elucidates the mechanism underlying the preventive effects of MSCs against pulmonary fibrosis.

## Data Availability

The data that support the findings of this study are available from the corresponding author upon reasonable request.

## Abbreviations

IPF: Idiopathic pulmonary fibrosis
EMT: Epithelial-mesenchymal transition
EndMT: Endothelial-to-mesenchymal transition
MSCs: Mesenchymal stem cells
ER: Endoplasmic reticulum
TGF-*β*1: Transforming growth factor-*β*1
UPR: Unfolded protein response
NAFLD: Nonalcoholic fatty liver disease
huMSCs: Human umbilical cord-derived mesenchymal stem cells
TM: Tunicamycin
CCK8: Cell Counting Kit 8
Q-PCR: Quantitative real-time PCR
HE: Hematoxylin and eosin
BLM: Bleomycin
HYP: Hydroxyproline
AEC: Alveolar epithelial cell.

## Supplementary Materials

Supplementary Materials Figure S1: A549 cells were treated with 10 ng/mL TGF-*β*1 in the presence or absence of MSCs for 72 hr. The protein expression levels of N-cadherin, TWIST1, Snail and Slug were measured using western blotting. Figure S2: Schematic of the mechanism by which MSCs decrease TGF-*β*1-induced EMT via inhibition of ER stress. TGF-*β*1 triggers the UPR and activates three critical ER stress-related pathways (the PERK/ATF4, IRE-1*α*/XBP1, and ATF6 pathways). Continuous ER stress contributes to EMT in A549 cells. MSCs ameliorate EMT by inhibiting ER stress, and modulation of the IRE-1*α*/XBP1 pathway plays a critical role in this process. MSC-mediated attenuation of EMT may be an important mechanism underlying the protective effect of MSCs against BLM-induced pulmonary fibrosis in mice. Table S1: primers used for Q-PCR.
