# Supplementary material for: Mesenchymal Stem Cells Inhibit Epithelial-to-Mesenchymal Transition by Modulating the IRE1α Branch of the Endoplasmic Reticulum Stress Response
Source: Stem Cells Int. 2023 Jul 26;2023:4483776. doi: 10.1155/2023/4483776 (PMC10397497; doi:10.1155/2023/4483776)
Supplement: Supplementary Materials — Figure S1: A549 cells were treated with 10 ng/mL TGF-β1 in the presence or absence of MSCs for 72 hr. The protein expression levels of N-cadherin, TWIST1, Snail and Slug were measured using western blotting. Figure S2: Schematic of the mechanism by which MSCs decrease TGF-β1-induced EMT via inhibition of ER stress. TGF-β1 triggers the UPR and activates three critical ER stress-related pathways (the PERK/ATF4, IRE-1α/XBP1, and ATF6 pathways). Continuous ER stress contributes to EMT in A549 cells. MSCs ameliorate EMT by inhibiting ER stress, and modulation of the IRE-1α/XBP1 pathway plays a critical role in this process. MSC-mediated attenuation of EMT may be an important mechanism underlying the protective effect of MSCs against BLM-induced pulmonary fibrosis in mice. Table S1: primers used for Q-PCR. [file 4483776.f1.docx]

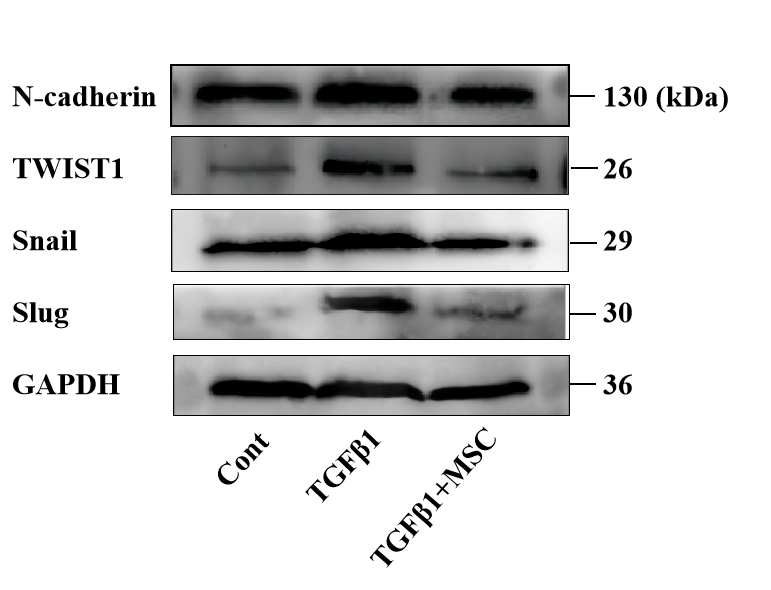


**Fig. S1.** A549 cells were treated with 10 ng/mL TGF-β1 in the presence or absence of MSCs for 72 h. The protein expression levels of N-cadherin, TWIST1, Snail and Slug were measured using Western blotting.


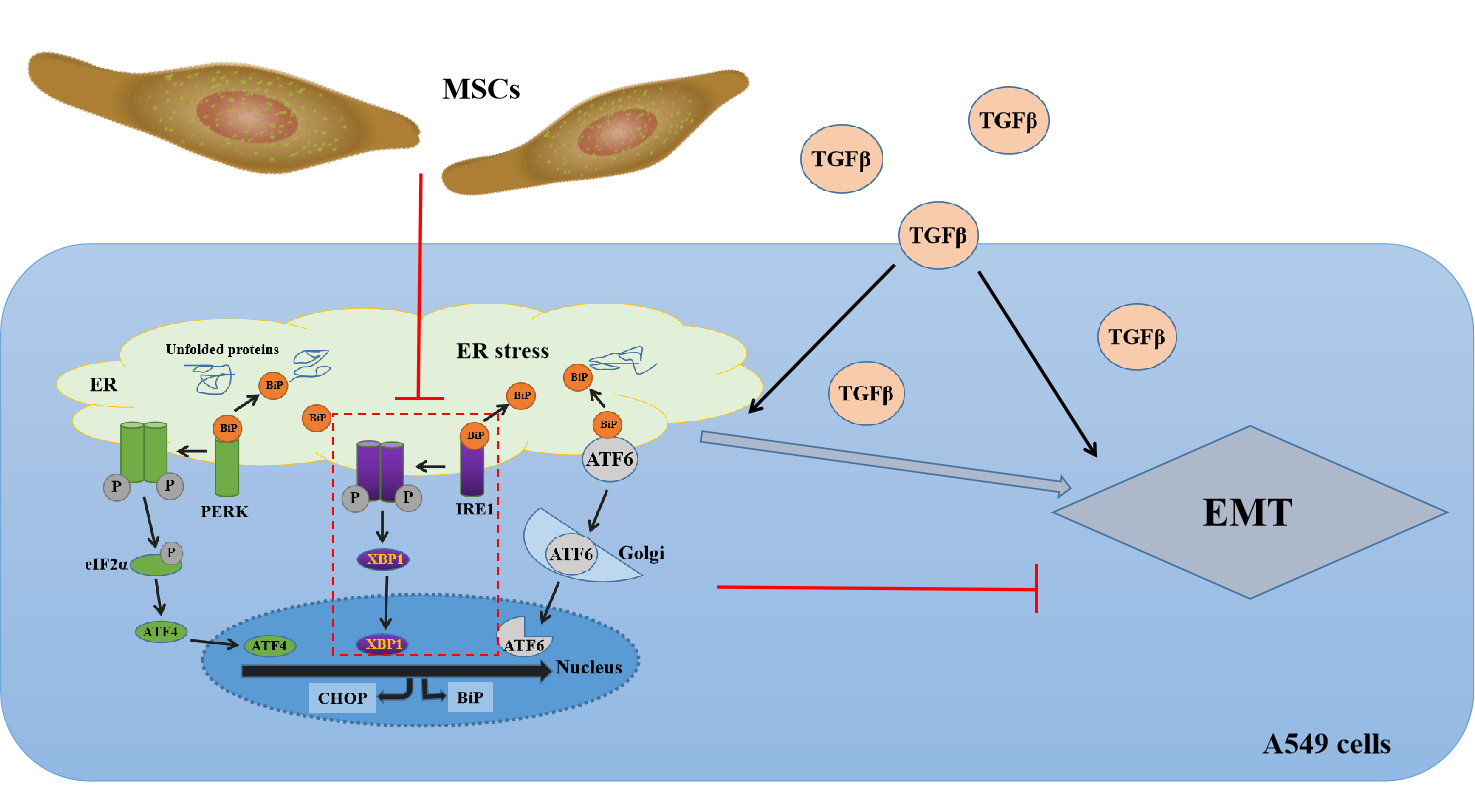


**Fig. S2.** Schematic of the mechanism by which MSCs decrease TGF-β1-induced EMT via inhibition of ER stress. TGF-β1 triggers the UPR and activates 3 critical ER stress-related pathways (the PERK/ATF4, IRE-1α/XBP1 and ATF6 pathways). Continuous ER stress contributes to EMT in A549 cells. MSCs ameliorate EMT by inhibiting ER stress, and modulation of the IRE-1α/XBP1 pathway plays a critical role in this process. MSC-mediated attenuation of EMT may be an important mechanism underlying the protective effect of MSCs against BLM-induced pulmonary fibrosis in mice.

**Table S1: Primers used for Q-PCR**

**Mouse**

| Gene | Forward | Reverse |
| --- | --- | --- |
| *Bip* | CTGATCGTTGGCTATGATCTCC | ATGATGAAGTTCACTGTGGTGG |
| *Chop* | CTCCAGATTCCAGTCAGAGTTC | ACTCTGTTTCCGTTTCCTAGTT |
| *Ire1* | GCAGGCTGTGTCTTTTACTATG | TATCAATTCACGAGCAATGACG |
| *Xbp1-s* | TTGCCTCTTCAGATTCTGAGTC | GGGGAAGGACATTTGAAAAACA |
| *E-cadherin* | CTCAGAAGACAGAAACGAGACT | AACCAGGTTCTTTGGAAATTCG |
| *Vimentin* | CAGCCTCTATTCCTCATCCC | GGTGTTCTTGAACTCAGTGTTG |
| *Atf6* | TGCCTTGGGAGTCAGACCTATGG | CTGTGGACCGAGGAGAGGAGATG |
| *Atf4* | AGTTTAGAGCTAGGCAGTGAAG | CATACAGATGCCACTGTCATTG |

**Human**

| Gene | Forward | Reverse |
| --- | --- | --- |
| *BiP* | AAGAACCAGCTCACCTCCAA | CACCTTGAACGGCAAGAACT |
| *CHOP* | GAGAATGAAAGGAAAGTGGCAC | ATTCACCATTCGGTCAATCAGA |
| *IRE1* | CGTGAGCGACAGAATAGAAAAG | GCTTCTTATTTCTCATGGCTCG |
| *XBP1-s* | CCCTCCAGAACATCTCCCCAT | ACATGACTGGGTCCAAGTTGT |
| *E-cadherin* | AGTCACTGACACCAACGATAAT | ATCGTTGTTCACTGGATTTGTG |
| *Vimentin* | ATGTCCACCAGGTCCGTGT | TTCTTGAACTCGGTGTTGATGG |
| *ATF6* | CTGATGGCTGTTCAATACACAG | GATCCCTTCGAAATGACACAAC |
| *ATF4* | ATGGATTTGAAGGAGTTCGACT | AGAGATCACAAGTGTCATCCAA |
